# Fluoroquinolone-Associated Disability: A Rodent Model Reveals Transient Neuropsychiatric and Persistent Gastrointestinal Effects of Low-Dose Ciprofloxacin

**DOI:** 10.3390/ph18091277

**Published:** 2025-08-27

**Authors:** Bitseat Getaneh, Jacqueline Kerler, Maral Ganzorig, Courtney Muhl, Alden Miller, Ini Efiom-Ekaha, Bethy Belai, Cecilia Bove

**Affiliations:** 1 Department of Pharmacology and Physiology, Georgetown University School of Medicine, Washington, DC 20007, USA; 2 Department of Biology and Marine Biology, College of Science and Engineering, University of North Carolina at Wilmington, Wilmington, NC 28403, USA; jek7740@uncw.edu; 3 Department of Biology, Kinsley School of Engineering, Sciences and Technology, York College of Pennsylvania, York, PA 17403, USA; mganzorig@ycp.edu (M.G.); cmuhl@ycp.edu (C.M.); 4 Department of Neuroscience, Dickinson College, Carlisle, PA 17013, USA; 5 Johns Hopkins Bloomberg School of Public Health, Baltimore, MD 21205, USA; 6 Milken Institute School of Public Health, George Washington University, Washington, DC 20052, USA; b.belai@gwmail.gwu.edu

**Keywords:** fluoroquinolones, FQAD, ciprofloxacin, gastrointestinal dysmotility, vagus nerve, anxiety, animal model

## Abstract

**Background/Objectives**: Fluoroquinolones (FQs) are broad-spectrum antibiotics associated with a constellation of severe, long-lasting adverse effects termed Fluoroquinolone-Associated Disability (FQAD), which often includes neuropsychiatric and gastrointestinal (GI) symptoms. Despite patient reports, GI dysfunction is not formally recognized within FQAD. This study aimed to establish a rodent model to investigate whether ciprofloxacin (CPX), the most commonly prescribed FQ, exposure induced long-lasting anxiety-like behavior and/or GI motility alterations. **Methods**: To test our hypothesis, Sprague Dawley rats were orally administered 20 mg/kg CPX, amoxicillin (AMX, antibiotic control), or saline (CTL) daily for 14 days. Anxiety-like behaviors were assessed weekly for 4 weeks post-treatment using the Elevated Plus-Maze, Marble Burying, and Open Field tests. GI transit was measured 2 weeks post-treatment via phenol red dye recovery analysis from the stomach and portions of the small intestine. **Results**: Our results demonstrated that CPX induced a transient, mild anxiety-like phenotype in rats, with behavioral changes largely resolving by week 4, becoming statistically indistinguishable from the CTL group. In contrast, CPX significantly accelerated GI transit, similar to the known prokinetic AMX, as evidenced by increased fractional dye recovery in the stomach and distal small intestine. This accelerated GI motility persisted weeks after CPX discontinuation. **Conclusions**: These findings establish a putative rodent model for FQAD, providing evidence that even small doses of CPX can induce acute, transient neuropsychiatric effects and, critically, persistent GI dysmotility. This supports the inclusion of GI dysfunction in FQAD symptomatology and highlights the need for judicious FQs prescription and comprehensive patient monitoring.

## 1. Introduction

Fluoroquinolones (FQs) are widely prescribed antibiotics. Their primary use is to treat serious bacterial infections when other antibiotics are ineffective. Recent data from the Centers for Disease Control and Prevention (CDC) shows that in 2022 the rate of prescription of FQs was 44 for every 1000 patients [1]. In Europe, with the exclusion of Cyprus and Germany, the defined daily dose (the assumed average maintenance dose per day for a drug used for its main indication in adults) was 0.15 and 1.16 DDD/1000 citizens/day for inpatient and outpatient respectively [2]. Despite the encouraging decline in the overall number of prescriptions in those two geographical regions, FQs are still often preferred due to their strong efficacy. Indeed, FQs are broad-spectrum antibiotics that work by inhibiting type-II DNA topoisomerases, and preventing bacterial mRNA transcription and DNA duplication from taking place [3,4,5].

In addition to common side effects, usually observed with the use of antibiotics [6], several side effects, including photosensitivity [7,8], tendon ruptures [9,10,11,12,13], psychiatric events [14,15,16,17,18,19,20,21,22], peripheral neuropathy [23,24], musculoskeletal and nervous system disability [25], aortic aneurysm and dissection [13,26,27,28,29,30], and aortic and mitral valve regurgitation [31] have been reported within the last two decades. Evidence suggests that these side effects are severe and potentially disabling, long-lasting, and most likely underreported by patients and clinicians [5,25,32,33]. The Food and Drug Administration (FDA) proposed the existence of a “Fluoroquinolone Associated Disability” (FQAD), which, while not yet formally recognized and assigned a diagnostic code, warns clinicians about the dangers of prescribing FQs due to the potential of harmful, long-term side effects [34]. First described by Debra Boxwell, FQAD is defined as a “constellation of disabling symptoms” which results in “a substantial disruption of a person’s ability to conduct normal life functions”, includes adverse events which “last 30 days or longer after stopping the fluoroquinolone”, from two or more of the following body systems: musculoskeletal, neuropsychiatric, peripheral nervous system, senses (vision, hearing, etc.), skin, and cardiovascular” [35]. Still, in 2018, it was reported that 19.9% of FQs prescriptions were given for infections for which FQs were not a recommended treatment [36].

Patient reports have suggested that FQs may lead to other side effects not recognized under FQAD, such as long-term disruption of normal gastrointestinal (GI) patterns. A recent publication from our group suggested that six different FQs were associated with a wide range of GI symptoms not currently reported in the drugs’ labels, including dyspepsia, dysphagia, pancreatitis (acute and chronic), constipation, gastritis, colitis, bloating, and persistent abdominal pain. Notably, the responses to the Bowel Disease Questionnaire administered to the study participants suggested that ~70% of FQs users scored positive for a functional gastrointestinal disorder, with no correlation between drug therapy and the symptoms reported [37]. This study highlights the need for more basic and clinical research on FQAD.

While the mechanism of FQs toxicity is not fully understood, many theories have been proposed, including inhibition of collagen production [10,13,38,39,40,41], increase in reactive oxygen species [4,19,29,42,43,44], and direct off-target effects in the Central Nervous System (CNS). Previous literature suggests that FQs act as selective antagonists of γ-aminobutyric acid (GABA), a major inhibitory neurotransmitter in the CNS, thereby disrupting GABAergic signaling and potentially causing CNS overactivation. Indeed, administration of 50 mg/kg, but not 20 mg/kg of ciprofloxacin (CPX), reduces GABA levels in brain tissue, which has been linked to symptoms like depression and anxiety [21]. In vitro studies have shown that FQs decrease the amplitude of GABA-mediated potentials in vagus nerve preparations, but not in the optic nerve, suggesting that the vagus nerve may be particularly susceptible to FQs [15,16]. Additionally, FQs enhance glutamatergic transmission through NMDA receptors in the rat hippocampus. This effect is thought to be related to structural similarities between FQs and kynurenic acid, an NMDA receptor agonist [17,18]. Experimental findings have demonstrated that pefloxacin induces seizures in mice, with alterations observed in both GABA and glutamate transmission [17]. Similar results were noted in a rat model of absence epilepsy following intraperitoneal injection of CPX [20]. The dual impact of FQs on these two key neurotransmitter systems in the CNS—reducing GABAergic activity and enhancing glutamate transmission—may contribute to the neurological and neuropsychiatric effects observed in FQAD.

The GI system is under the modulation of the vagus nerve, a key component of the parasympathetic nervous system [45,46,47]. Through the vago-vagal reflex, a variety of neurotransmitters and neuromodulators, mainly GABA and glutamate, are released from the Nucleus of the Tractus Solitarius in response to peripheral gastrointestinal signals to regulate the release of acetylcholine (ACh) from preganglionic parasympathetic neurons within the Dorsal Motor Nucleus of the vagus (DMV) [48,49,50,51,52,53]. ACh release leads to the activation of postganglionic myenteric neurons in the GI tract, which belong to the excitatory cholinergic or inhibitory non-adrenergic non-cholinergic pathways to elicit or prevent contractions of the GI tract from occurring [54,55,56,57].

Interestingly, other effects of FQs on the CNS, such as anxiety and depression [58,59,60], can be correlated with the vagus nerve. For instance, the vagus nerve communicates with the locus coeruleus (LC), the primary source for norepinephrine in the CNS. A dysregulated vagus nerve may translate into a dysregulated LC, whose neurons are activated to encode fear, anxiety, and have been associated with anxiety, insomnia, or depressive-like states [61]. Furthermore, vagus nerve stimulation (VNS) was approved for treatment-resistant depression, and is more effective than conventional antidepressants [62], implying a role of the vagus in the pathophysiology of this mood disorder. Interventions to regulate vagal tone were proven successful in reducing anxiety in young and older adults [63]. Whether these effects are caused by a reduction in neuroinflammation through VNS [64] or by a direct impact of the brain-gut axis on higher CNS centers [65] remains unclear.

The goal of this study was to test A) whether 20 mg/kg of CPX would induce long-lasting anxiety-like behavior even after discontinuation of the antibiotic and B) whether the same dosage would induce an alteration in GI motility. The effects of CPX were tested against saline (CTL) and amoxicillin (AMX). By monitoring anxiety-like symptoms at baseline and every 7 days for a total of 4 weeks, and by measuring GI transit at 2 weeks post-treatment, we observed a transient increase in anxiety symptoms with CPX, only lasting for the duration of the antibiotic treatment, and an acceleration of GI dysmotility persisting even after discontinuation of the antibiotic. While the pathophysiological mechanism behind these side effects was not investigated in this study, we provide evidence for including GI disorders in the constellation of symptoms of FQAD and a model that could help identify a clearer mechanism of disease for this syndrome.

## 2. Results

### 2.1. Ciprofloxacin Only Causes a Transient Anxiety-like Phenotype

To evaluate the possibility that CPX can lead to the development of anxiety-like behaviors in rats at 20 mg/kg, a battery of behavioral tests was administered at baseline and every week thereafter for a total of 5 weeks. Their behavior was analyzed against a negative control and a positive control, the antibiotic AMX. This antibiotic, which is the most commonly prescribed worldwide, was chosen as an additional control group to determine whether the effects observed in the behavioral tests are due to antibiotic treatment in general or if they are specific to the mechanism of action of FQs. Moreover, AMX is known to cause acceleration of GI motility and can serve as a positive control in that regard [66,67].

**Figure 1:**
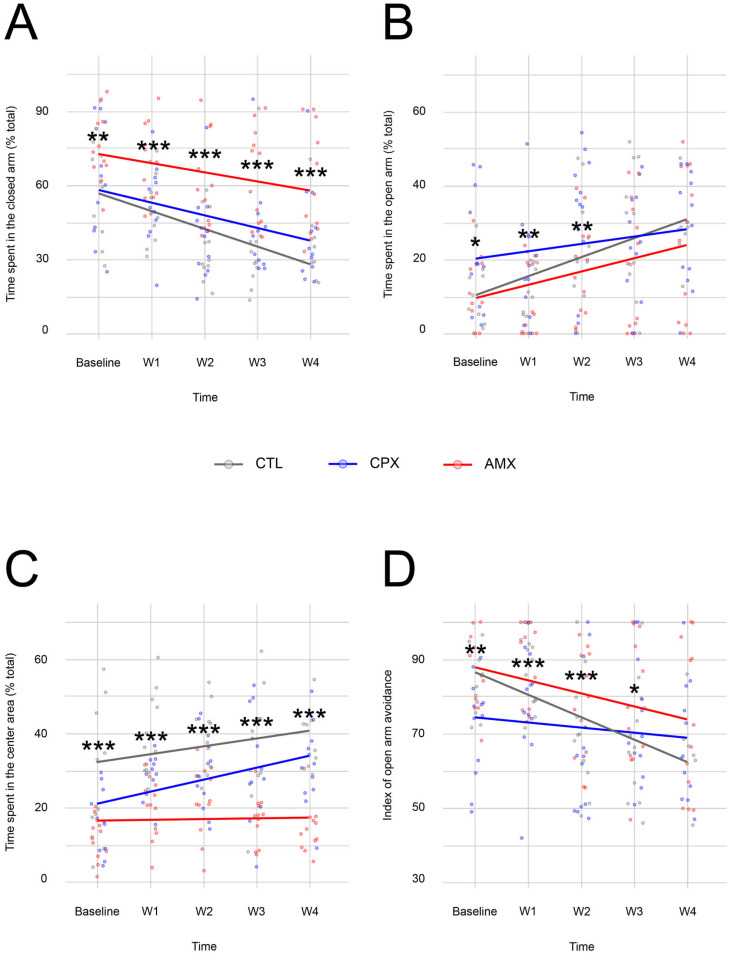
(**A**) The Linear Mixed Model showed that CTL- (gray) and CPX-treated animals (blue) spent significantly less time in the closed arm compared to the AMX group (red) at all time points. No significant difference was observed between CTL and CPX at any time point. (**B**) CPX-treated animals showed statistically significant differences from the AMX and CTL groups at baseline, and from AMX during the first two weeks of treatment in the amount of time spent in the open arm. Notably, CPX-treated animals displayed a slower increase in the amount spent in the open arm. (**C**) CTL- and CPX-treated animals showed an increasing preference for the center neutral square, whereas the AMX group exhibited minimal differences. Significant differences between all groups were observed at all time points. (**D**) In agreement with the percentage of time spent in the open and closed arms, animals from all groups had a reduction in the index of open arm avoidance with time, with CPX showing a smaller decline over time. Significant differences were observed between all groups at baseline and week 1, with this difference disappearing after the end of the treatment at week 2. *p* < 0.05 (* = 0.01 < *p* < 0.05; ** = 0.001 < *p* < 0.01; *** = 0 < *p* < 0.001). *n* = 6–12 for CTL, *n* = 12 for CTL and AMX.

In the Elevated Plus-Maze Test, animals in all groups showed a downward trend in the percentage of time spent in the closed arm, with the CTL group spending the least time in the closed arm at the last time point (35.22 ± 5.05% of total time), and CPX-treated animals falling between the CTL and the AMX group (40.14 ± 5.73% and 59.93 ± 6.34% of total time for CPX and AMX respectively; Figure 1A). The LMM suggests that the differences observed between the groups are due to the saline or CPX treatments and time separately. Indeed, CTL and CPX-treated animals appeared to spend significantly less time in the closed arm compared to the AMX (antibiotic control) group at all time points tested; however, no difference seemed to exist between the CTL and the CPX group at any time point.

In correlation with the closed arm data, all groups showed an increase in time spent in the open arm. Similarly, CPX animals fell between the comparison groups (25.17 ± 4.3%, 28.09 ± 4.45%, and 24.67 ± 5.89% of total time for CTL, CPX and AMX respectively at week 4; Figure 1B). The model indicated that CPX and time affected the behavioral response separately; however, the difference is statistically significant at baseline (CPX versus AMX and CTL), and during the first two weeks only, when the treatments were ongoing (CPX= 52.84 ± 4.54% at week 1 and 40.66 ± 5.83% at week 2 vs. AMX= 67.62 ± 4.58 at week 1 and 59.58 ± 5.48% at week 2).

The observed increase in time spent in the open arm is correlated with an increase in time spent in the central neutral square, except for AMX animals, who showed virtually no changes in their behavioral output (Figure 1C). The LMM suggested that the statistically significant differences between the groups are dependent on the saline treatment and the interaction between CPX treatment and time. Specifically, post-hoc analysis indicated that there is a difference between all groups at baseline; following that time point, all groups continued showing a difference in the percentage of time spent in the neutral square, except for week 4, where CTL and CPX animals behaved similarly (39.63 ± 2.61%, 31.76 ± 3.12%, and 14.99 ± 1.82% of total time for CTL, CPX and AMX respectively).

To understand whether the number of entries in the two arms contributed to the changes in time spent in those two areas, an index of open arm avoidance was calculated as described in the methods. The data summarized in Figure 1D showed a downward trend for all groups, with CTL animals having the lowest index at the final time point and the steepest slope (65.55 ± 5.33, 68.79 ± 4.43, and 73.57 ± 5.92 for CTL, CPX and AMX respectively at week 4). The model suggested an effect from CPX and time separately, with all groups showing different behavioral outputs at baseline and week 1 (86.12 ± 2.69, 78.03 ± 4.36, and 89.5 ± 3.28 for CTL, CPX and AMX respectively at week 1). Statistically significant differences were observed at weeks 2 (71.5 ± 4.45, 75.7 ± 9.43, and 77.36 ± 4.26 for CTL, CPX and AMX respectively) and 3 (67.63 ± 4.93, 72.97 ± 4.77, and 77.63 ± 5.17 for CTL, CPX and AMX respectively) between CTL and CPX versus AMX animals. However, the difference between CPX and AMX was no longer significant by week 4 (Table 1).

Overall, the data suggest that CPX may induce a mild, short-term, anxiety-like state in the initial phase of administration of the drug, which did not appear to be long-lasting. Across multiple measures (open arm time, closed arm time, and avoidance index), the behavior of the CPX-treated group became statistically indistinguishable from the untreated CTL group by the end of the four-week experiment.

To assess the compulsive behaviors often associated with anxiety, the Marble Burying Test was also administered.

**Figure 2:**
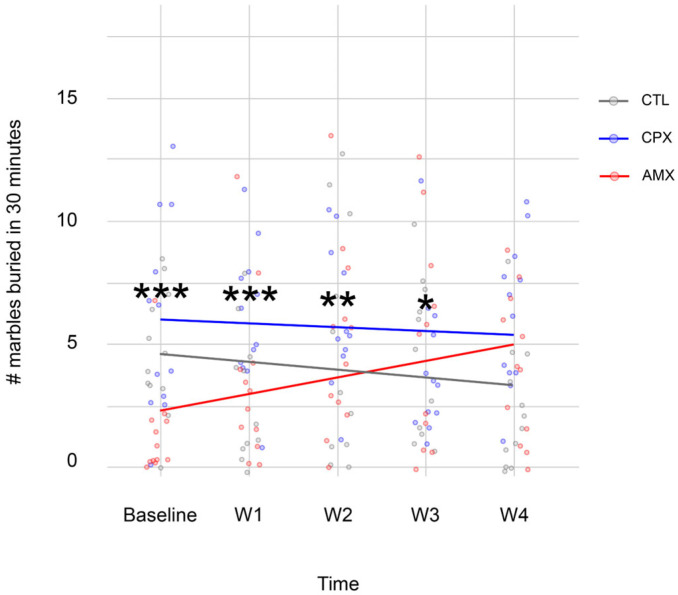
While CTL animals (gray) learned over time that there is no threat to their safety, the Linear Mixed Model showed that CPX animals (blue) stayed at baseline levels and had no improvement, suggesting an anxiety-like phenotype. Interestingly, AMX animals (red) increased the number of marbles buried with time. Nonetheless, no statistically significant difference was observed at the final time point. *p* < 0.05 (* = 0.01 < *p* < 0.05; ** = 0.001 < *p* < 0.01; *** = 0 < *p* < 0.001). *n* = 12 for all groups.

Figure 2 shows the number of marbles buried during the 5 trials analyzed. As expected, CTL animals buried fewer marbles with time (5.92 ± 0.92 at baseline vs. 3.08 ± 0.95 at week 4), indicating a lack of preoccupation for their safety; CPX animals stayed at baseline (7.67 ± 1.52 at baseline vs. 7.92 ± 1.14 at week 4); finally, AMX animals showed a behavior opposite to CTL (1.58 ± 0.74 at baseline vs. 5.17 ± 1.10 at week 4). The model predicted a strong interaction between each treatment and time (t-values = 3.685 for CPX; 2.278 for CTL; 2.368 for time; −2.041 for CPX and time; −1.2833 for CTL and time. *p*-values = <0.05 for all). The Bonferroni post-hoc analysis shows that CPX is significantly different from all groups at every time point observed, except for the final week.

These results aligned with the Elevated Plus-Maze Test, suggesting that CPX had a transient effect on the behavioral output.

Finally, to confirm the results described above, the Open Field Test was also administered.

**Figure 3:**
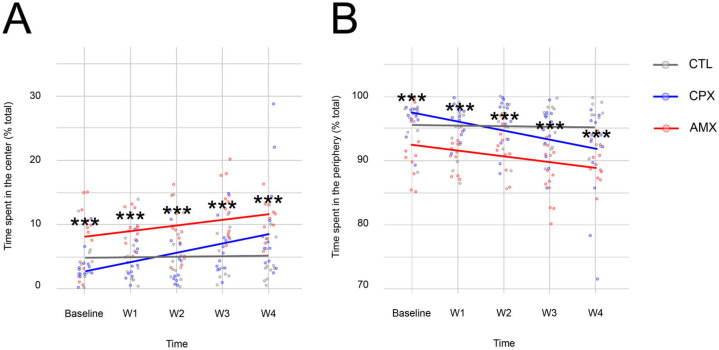
The Linear Mixed Model showed that compared to CTL-treated animals (gray), rodents in the CPX group (blue) displayed an increased preference over time for exploration in the center area (**A**) and a reduced preference for the periphery (**B**) at the final time point, which did not provide additional evidence of the transient nature of the anxiety phenotype observed in the Elevated Plus-Maze and Marble Burying Tests. AMX-treated animals (red) showed a statistically significant difference from the other groups at all time points. *p* < 0.05. (*** = 0 < *p* < 0.001). *n* = 12 for all groups.

While CTL animals did not display any changes in the percentage of time spent in either the center (Figure 3A) or the periphery (Figure 3B) with time, CPX- and AMX-treated animals spent progressively more time in the center and less time in the periphery, with some differences between all groups observed at baseline.

The model indicated that saline, CPX, and time affected the behavioral output. While AMX-treated animals behaved differently from all groups at all time points, there is a statistically significant difference between CTL (4.1 ± 0.87% of total time) and CPX (9.532 ± 0.33% of total time) at week 4 in the time spent in the center only, where it appeared that CPX animals had a significantly higher preference for the center area compared to the periphery of the open field. Analysis of the time spent in supported versus unsupported rearing did not show any differences between the groups at any time point, the data is summarized in Table 2.

This data shows that at 20 mg/kg, CPX did not support the results of the Elevated Plus-Maze and Marble Burying Tests.

### 2.2. Ciprofloxacin Accelerates Transit Similarly to Amoxicillin

To evaluate whether CPX at 20 mg/kg affects GI transit, phenol red dye was administered via oral gavage, and the stomach and the small intestine were harvested post-mortem 2 weeks after the end of the treatment with saline, CPX or AMX. The amount of dye recovered from each segment of the GI tract was measured via spectrophotometry to indirectly assess GI motility.

**Figure 4:**
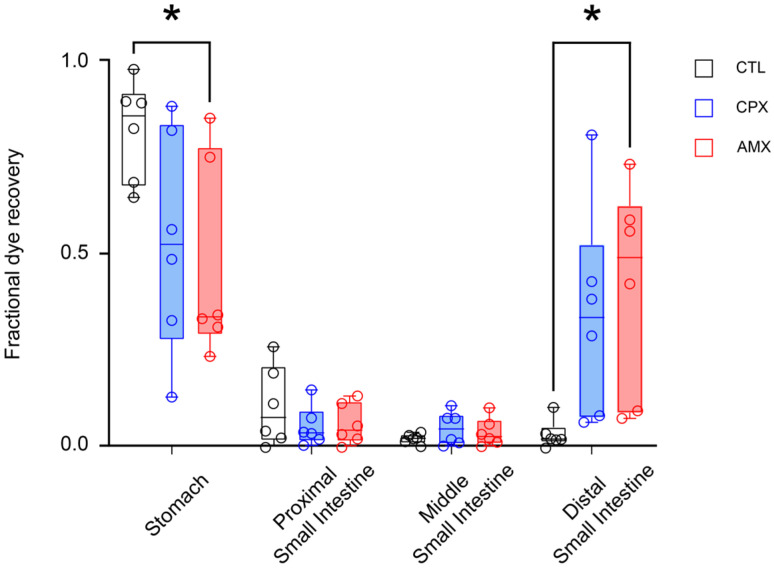
Box-whisker plot representing the fraction of dye recovered from each GI segment in CTL- (gray), CPX- (blue), and AMX-treated animals (red; *n* = 6 for all). The data showed that, in the CTL group, most dye was recovered from the stomach. Virtually no dye was recovered in the other segments. As expected, more dye was recovered in the distal small intestine in the AMX group. Similarly, more dye was detected in the same segment in CPX-treated animals. *p* < 0.05 (* = 0.01 < *p* < 0.05).

Figure 4 shows that, compared to saline-treated animals, treatment with CPX caused a reduction in the fraction of phenol red recovered from the stomach (0.542 ± 0.118), which correlates with an increase in dye found in the distal small intestine (0.348 ± 0.113). Interestingly, CPX-treated animals displayed a GI motility virtually indistinguishable from that of AMX-treated animals, from which we expected a marked acceleration of GI transit. Indeed, there is a statistically significant difference between CTL- and AMX-treated animals in the fractional dye recovery from the stomach (0.830 ± 0.054 and 0.477 ± 0.107 for CTL and AMX, respectively) and distal small intestine (0.036 ± 0.015 and 0.418 ± 0.112 for CTL and AMX, respectively).

This data suggests that CPX causes an acceleration in GI transit similar to AMX, which has known prokinetic properties, even 2 weeks after the end of the treatment.

## 3. Discussion

This study aimed to investigate whether 20 mg/kg CPX would induce long-lasting anxiety-like behavior and alterations in GI motility. At a dose of 20 mg/kg, CPX induced a transient, mild anxiety-like phenotype in rats, as evidenced by changes in the Elevated Plus-Maze, Marble Burying, but not the Open Field Tests. It is important to note that these behavioral changes were largely resolved by week 4, becoming statistically indistinguishable from the CTL group. However, at the same time point, CPX accelerated GI transit in a manner similar to the animals treated with the prokinetic AMX, as evidenced by the increased fractional dye recovery in the stomach and distal small intestine. 

The behavioral data partially support previously published data showing that CPX can induce anxiety-like symptoms after 14 days of daily administration; however, the study by Ilgin and collaborators observed said symptoms in the Elevated Plus-Maze test only, using a higher dosage of the drug (50 mg/kg) and a different model (female Wistar rats) [21]. Our data extends their findings by demonstrating an effect even at a lower dose, though it appears to resolve with discontinuation of the drug treatment. This transient anxiety may be due to FQs’ effects on GABAergic and glutamatergic transmissions as discussed earlier in the manuscript [14,15,16,17,18,20,21]. Understanding whether the temporary nature of the symptoms is due to clearance of the drug from the system or engagement of compensatory mechanisms in the CNS could be important for the management of psychiatric symptoms associated with FQAD. Interestingly, AMX treatment showed contrasting or less pronounced behavioral effects. While some studies showed that this antibiotic can cause depression and anxiety in mice [68,69], we did not observe any statistically significant alteration in behavior, suggesting that the changes observed at the week 2 time point are specific to CPX rather than a general effect of antibiotic treatment on the organism.

The dose of CPX used in this study caused a decrease in the fraction of phenol red recovered from the stomach and an increase in the distal small intestine, suggesting that CPX caused an acceleration in the movement of contents through the GI tract. This finding supports our previously published study in which we correlated the use of different FQs to self-reported symptoms matching the diagnosis for functional gastrointestinal disorders even in participants who interrupted the antibiotic treatment after one dose [37]. Considering the predominant role of the vagus nerve in regulating GI motility [45,46,47,49], the data shown in this manuscript are a strong indication that the vagus nerve is a plausible target of FQs. It is important to remember that previous literature demonstrated that treatment with FQs caused inhibition of GABAergic receptor activity on ex vivo vagus nerve preparations, which correlated to an increase in nerve activity [16]; although we did not directly measure vagal activity, this study offers a strong confirmation of the findings by Green and Halliwell. Interestingly, the observed acceleration in transit is statistically identical to that caused by AMX, a known prokinetic [66,67]. Further investigation is necessary to determine whether the observed acceleration in GI motility is due to a broader antibiotic effect, such as dysbiosis, or both drugs impacting this system through distinct pathways. Nonetheless, since the accelerated transit was observed after discontinuation of the treatment, when all CPX would be eliminated from the system [70], our findings support the idea of including prolonged, if not permanent, GI dysmotility as part of the constellation of symptoms of FQAD.

The evidence of GI dysmotility at week 4, when behavioral symptoms disappeared, could be explained by a variety of mechanisms. First, and possibly most straightforward explanation, is that the vagus nerve does not contribute to the anxiety-like phenotype reported herein. While some evidence exists in support of that idea [61,63,64,65], it is possible that this is not the case with FQs, and that the two side effects are not correlated in FQAD. Alternatively, we may be observing a threshold-like effect, where the peripheral component of the vagus becomes affected before the central component of the vagus and other CNS centers. This was reported in neurodegenerative diseases such as Parkinson’s Disease, where GI dysfunction appears decades before the onset of symptoms associated with impairment of higher CNS centers [71,72,73]. Further studies observing the direct impact of FQs on the electrophysiological activity of the DMV, or observing the impact of FQs on anxiety-like behavior and GI dysmotility in vagotomized rats, may be important to elucidate the mechanism.

This study establishes a relatively easy-to-replicate, putative model of FQAD at a dose that can successfully reproduce the FQs-induced anxiety (on an acute scale) and that strongly suggests an involvement of the GI tract in FQAD. Considering the vast array of symptoms associated with this syndrome [5,33], this model may be invaluable for future mechanistic studies aimed at elucidating the complex pathophysiology behind this understudied condition. Additionally, this model may be helpful to explore therapeutic interventions. Furthermore, there is a possibility that the transient nature of the anxiety-like phenotype reported herein could be the result of a dose-dependent onset of the symptoms. In support of this idea, the study by Ilgin reported several alterations in neurotransmitter levels correlated to mood dysregulation only at the 50 mg/kg dose [21]; this is a critical piece of information in our understanding of FQAD and the spectrum of the symptoms reported by patients. Judicious FQs prescription beyond the known black box warnings should become the norm even for patients currently considered as “low risk”.

This study has its limitations. To start, only one dose was investigated (20 mg/kg). Studying higher doses or prolonged exposure beyond the 14 daily administration regimen could perhaps show more persistent or dramatic neuropsychiatric and/or GI symptoms. More importantly, the study does not elucidate the precise mechanism behind these symptoms. A plethora of factors could contribute to the putative vagal dysfunction described herein. Specifically, altered GABAergic and glutamatergic neurotransmission between the NTS and the DMV could cause an increase in vagal nerve activity resulting in the observed GI transit [16,33,49]. At the same time, the possibility of disrupted ACh or NO release from the myenteric neurons regulated by the vagus nerve should be investigated [54,55,56,57]. Finally, vagal activity is strongly influenced by circulating levels of various digestive hormones, including but not limited to cholecystokinin and ghrelin [74,75,76,77]. While CPX has been shown to acutely affect vagal activity in ex vivo vagus nerve preparations [16], it would be fascinating to measure the electrophysiological properties of this nerve or associated medullary nuclei isolated from animals that received CPX as treatment. Finally, this study does not establish a direct causal link or shared mechanism between the observed transient anxiety and GI dysmotility upon CPX exposure. All these areas should be investigated in future studies to deepen our understanding of FQAD.

## 4. Materials and Methods

### 4.1. Ethics Statement

The procedures in this experiment were approved by the Institutional Animal Care and Use Committee following Bucknell University’s and York College of Pennsylvania’s guidelines for animal research.

### 4.2. Animals and Treatment

Sprague Dawley rats of both sexes of approximately 30 days of age were housed in groups of three in an Animal Care Facility maintained at 25 °C on a 12:12 light-dark cycle. The animals had ad libitum access to food and water.

Rats were randomly divided into three groups: (1) a negative control (CTL), (2) an antibiotic control (AMX), and (3) an experimental group (CPX). Animals in each group were administered an oral gavage of either 0.3 mL of 0.9% saline solution (CTL) or 20 mg/kg of body weight of amoxicillin (AMX) or ciprofloxacin (CPX) daily for 14 days (*n* = 12 for all groups). The dosage for CPX was chosen based on previously published studies in which 20 mg/kg were used [21,78], without causing cardiotoxicity [79]. To keep stress at a minimum for the animals, we opted for a once daily, comparable dosage of amoxicillin as described in the literature [80].

### 4.3. Behavior

Each behavioral test described below was conducted for all groups at baseline (i.e., before the beginning of the treatment) and every week thereafter for a total of five consecutive weeks. Animals were allowed to acclimate in the testing room for at least one hour before the beginning of the experiment. No more than one behavioral test was performed daily, for a total of three behavioral tests per week. Animals were excluded from a time point if they failed to complete the task (i.e., lack of exploration of the open field/escape attempts, or escape from the Elevated Plus-Maze).

The battery of behavioral tests used in this study includes:

#### 4.3.1. The Elevated Plus Maze Test

The Elevated Plus Maze Test: this test was conducted based on previous literature recommendations [81]. Briefly, the experiments were conducted during the dark phase of their light: dark cycle in a dedicated room with a singular source of light pointed at the center of the maze set to 30 lux. During the test, the rats were placed in the maze’s central square, facing one side of the open arms, and allowed to freely move into any of the arms for 5 min. The rodent’s activity was video-recorded for analysis. The time spent in the open arms, closed arms, or center square was noted and expressed as a percentage of the total time in the maze. The number of entries in the closed or opened arms was recorded to calculate the index of open arm avoidance as described by Trullas and Skolnick [82]:

$$100-\frac{\left(\%\;open\;arm\;entries+time\;spent\;in\;open\;arms\right)}{2}$$

#### 4.3.2. The Marble Burying Test

The Marble Burying Test: more details on this test can be found in previously published literature [83]. Briefly, the test was conducted during the light phase of the animals’ light:dark cycle. Standard polycarbonate rat cages (26 cm × 48 cm × 20 cm) with fitted filter-top covers were filled with 5 cm of unscented bedding material. 20 glass marbles were placed at equal distances of 5 rows × 4 columns on top of the bedding. To test, the rat was placed in a corner of the cage as far away from the marbles as possible. During the test, rats had no access to food and water. The rats were gently removed from the cage after 30 min, and the number of buried marbles was counted. Marbles were considered buried when at least two-thirds of their surface was covered with bedding material.

#### 4.3.3. The Open Field Test

The test was conducted as described previously [84]. Briefly, the experiments were conducted during the dark phase of their light: dark cycle. The Open Field Test apparatus consisted of a 50 cm × 50 cm × 38 cm acrylic box. The floor of the box was marked with a grid to distinguish the center from the outside areas, closer to the walls of the box. The center area was broadly illuminated to approximately 50 lux. At the beginning of the test, each rat was placed in the center area and allowed to move freely for 10 min. The time spent in the center area and the time spent at the periphery were recorded and expressed as a percentage of the total time in the box. Time spent in supported versus unsupported rearing was also recorded and expressed as a percentage of the total time in the field, and then expressed as a ratio (supported rearing/unsupported rearing).

### 4.4. Gastrointestinal Transit

To assess GI transit, we used a procedure described before [85]. Briefly, 2 weeks following the last day of the oral treatment the rodents were fasted overnight with only access to water. On the experimental day, the rats were fed an oral gavage of 1.5 mL of a 5% glucose solution containing 0.5 mg/mL of phenol red dye (Sigma Aldrich, Merck KGaA, Darmstadt, Germany). 1 h after the gavage was administered, rats were humanely sacrificed via anesthetic overdose (Isofluorane, Patterson Veterinary, Loveland, CO, USA). The entire stomach, 40% of the proximal small intestine (PSI), and 30% of the middle (MSI) and distal (DSI) small intestines were harvested. Volume displacement from these portions was measured and recorded. The tissues were homogenized in a 0.1N NaOH solution and allowed to sediment for 20 min. 10 mL of the supernatant was collected and centrifuged at 2800 rpm for 10 min. Protein residues were precipitated by adding 0.5 mL of trichloroacetic acid to 5 mL of the resulting supernatant. Following another round of centrifugation, 3 mL of the resulting supernatant was collected and suspended with 4 mL of a 0.5 N NaOH solution. The phenol red dye recovered in the supernatant was then measured via spectrophotometry at 560 nm. Fractional dye recovery was calculated as follows:

$$FDR\left(\%\right)=\;\frac{C\;\times \;volume}{mtot}$$

where C = absorbance recorded at the spectrophotometer, volume = volume displaced by the GI tract segment, and mtot = total absorbance from all segments.

Considering that high estrogen levels have been linked to slower GI motility in rodents [86], GI transit in female rats was only tested during the diestrus and metestrus phases of their estrus cycle, when estrogen levels are low. As such, any possible changes in GI motility observed could only be attributed to the drug treatments. To assess the estrus cycle, vaginal smears were collected the day of the experimentation as described by Jiang [86]. A cotton-tipped applicator soaked in 0.9% saline was inserted 1 cm into the vagina. The cells collected were smeared onto glass slides and observed microscopically. The estrus cycle phase was defined following the identification of the predominant cell type. Male animals were swabbed across the external perineum to control for handling.

### 4.5. Data Analysis

The number of animals needed to obtain statistically significant results was determined by performing power analysis for the behavioral studies and the gastrointestinal transit study (data available upon request).

The behavioral tests were analyzed using R statistical software (version 4.5.1; R Core Team, 2025) within RStudio (version 2025.05.1, Posit Team, 2025). Data manipulation and visualization utilized the following packages: “readr”, “dplyr”, “lme4”, “lmerTest”, “ggplot2”, “emmeans”, “tibble”, and “tidyr” [87,88,89]. A Linear Mixed-Effects Model (LMM) was employed to assess the effects of the treatment, time, and their interaction on the behavioral output. The subject ID was included as a random intercept to account for repeated measures within individual animals. For the group explanatory variable, Amoxicillin was used as the reference level because they were alphabetically first. The model structure was defined as:

Behavioral Score ~ Group × Time + (1|Subject_ID)

The goodness of fit of the model was checked by plotting residuals vs. fitted values and by comparing the quantiles of the model’s residuals against the quantiles of a theoretical normal distribution (Q-Q plot). Following the overall model, estimated marginal means (EMMs) were calculated for each treatment at each time point. Pairwise comparisons between treatments at each time point were then performed and adjusted for multiple comparisons using the Bonferroni method for 3 tests.

The fractional dye recovery experiment was analyzed using GraphPad Prism (GraphPad Software, version 10.5.0, 2025) with a One-Way ANOVA to compare the amount of dye recovered in each segment of the GI tract between the three groups. Post-hoc comparison was performed using Tukey’s method.

Data was considered significant if *p* < 0.05.

## 5. Conclusions

In summary, this study provides evidence that GI dysmotility should be included in the constellation of symptoms of FQAD, and a potential rodent model with potential for pathophysiological and therapeutic studies in the context of this syndrome. Increased awareness of this syndrome should become the norm in clinical practice, even for prescriptions of low-dose FQs or for individuals not considered part of a vulnerable cohort. A more comprehensive assessment of patients receiving this important class of antimicrobials is encouraged, with particular emphasis on GI symptoms and early changes in anxiety to facilitate early identification and mitigation of FQAD symptoms.

## Figures and Tables

**Table 1:** Output for the Linear Mixed Model generated for the Elevated Plus-Maze Test. AMX was the variable (fixed effect) against which other parameters were tested. Statistically significant *p*-values are shown in bold.

Open Arm		
	t-value	*p*-value
Intercept	2.746	**0.008**
CPX	2.574	**0.011**
CTL	0.200	0.842
Time	2.971	**0.003**
CPX and time	−0.973	0.332
CTL and time	0.822	0.412
Closed arm		
	t-value	*p*-value
Intercept	16.457	**<2^−16^**
CPX	−2.817	**0.005**
CTL	−2.984	**0.003**
Time	−2.422	**0.017**
CPX and time	−0.740	0.460
CTL and time	−1.657	0.100
Central Neutral Square		
	t-value	*p*-value
Intercept	7.044	**2.37^−10^**
CPX	1.415	0.159
CTL	4.806	**3.55^−6^**
Time	0.195	0.846
CPX and time	2.339	**0.021**
CTL and time	1.441	0.152
Index of Open Arm Avoidance		
	t-value	*p*-value
Intercept	24.544	**<2^−16^**
CPX	−3.139	**0.002**
CTL	−0.287	0.774
Time	−2.913	**0.004**
CPX and time	1.245	0.215
CTL and time	−1.326	0.187

**Table 2:** Linear Mixed Model generated for the Open Field Test using AMX as comparison for the other groups. Statistically significant *p*-values are shown in bold.

Center		
	t-value	*p*-value
Intercept	7.796	**2.22^−10^**
CPX	−4.659	**6.57^−6^**
CTL	−2.895	**0.004**
Time	2.679	**0.008**
CPX and time	1.138	0.257
CTL and time	−1.748	0.082
Periphery		
	t-value	*p*-value
Intercept	91.535	**<2^−16^**
CPX	4.558	**1.01^−5^**
CTL	2.735	**0.007**
Time	−2.801	**0.006**
CPX and time	−1.142	0.255
CTL and time	1.847	0.067

## Data Availability

The original contributions presented in this study are included in the article. Further inquiries can be directed to the corresponding author.

## Abbreviations

The following abbreviations are used in this manuscript:

FQs	Fluoroquinolones
CDC	Centers for Disease Control and Prevention
FDA	Food and Drug Administration
FQAD	Fluoroquinolone Associated Disability
GI	Gastrointestinal
CNS	Central Nervous System
GABA	γ-aminobutyric acid
CPX	Ciprofloxacin
NMDA	(N-methyl-D-aspartate)
ACh	Acetylcholine
DMV	Dorsal Motor Nucleus of the vagus
LC	Locus Coeruleus
VNS	Vagus Nerve Stimulation
CTL	Control
AMX	Amoxicillin
PSI	Proximal Small Intestine
MSI	Middle Small Intestine
DSI	Distal Small Intestine
LMM	Linear Mixed effects Model
EMMs	Estimated Marginal Means
